# FANCD2 Confers a Malignant Phenotype in Esophageal Squamous Cell Carcinoma by Regulating Cell Cycle Progression

**DOI:** 10.3390/cancers12092545

**Published:** 2020-09-07

**Authors:** Lisa Chan Lei, Valen Zhuoyou Yu, Josephine Mun Yee Ko, Lvwen Ning, Maria Li Lung

**Affiliations:** Department of Clinical Oncology, University of Hong Kong, Hong Kong (SAR), China; u3004461@connect.hku.hk (L.C.L.); zvyu@hku.hk (V.Z.Y.); joko@hku.hk (J.M.Y.K.); u3003773@connect.hku.hk (L.N.)

**Keywords:** FANCD2, esophageal squamous cell carcinoma, cell cycle, ubiquitination

## Abstract

**Simple Summary:**

Fanconi anemia patients with germline *FANCD2* defects are susceptible to cancers. In order to understand *FANCD2* function in esophageal cancers, we used cell line, animal model, and sequencing approaches. We knocked out the *FANCD2* gene and examined the functional impact of its loss on tumor growth and metastasis and performed assays for cell growth, cell cycle, and cellular localization. *FANCD2* promotes tumorigenesis in this cancer. *FANCD2* is significantly upregulated in tumors. Depletion of FANCD2 protein expression significantly suppresses the cancer cell proliferation and tumor colony formation and metastasis potential, as well as cell cycle progression, by involving specific cell signaling pathways. FANCD2 is moved out of the nucleus to the cytoplasm during cell cycle progression. We provide evidence of a novel role of *FANCD2* in esophageal cancer progression and its potential value as a biomarker for disease management.

**Abstract:**

Fanconi anemia patients with germline genetic defects in *FANCD2* are highly susceptible to cancers. Esophageal squamous cell carcinoma (ESCC) is a deadly cancer. Little is known about the function of *FANCD2* in ESCC. For detailed molecular and mechanistic insights on the functional role of *FANCD2* in ESCC, in vivo and in vitro assays and RNA sequencing approaches were used. Utilizing Clustered Regularly Interspaced Short Palindromic Repeat (CRISPR) technology, *FANCD2* knockout models were established to examine the functional impact in mouse models for tumor growth and metastasis and in vitro assays for cell growth, cell cycle, and cellular localization. Our RNA sequence analyses were integrated with public datasets. *FANCD2* confers a malignant phenotype in ESCC. *FANCD2* is significantly upregulated in ESCC tumors, as compared to normal counterparts. Depletion of FANCD2 protein expression significantly suppresses the cancer cell proliferation and tumor colony formation and metastasis potential, as well as cell cycle progression, by involving cyclin-CDK and ATR/ATM signaling. FANCD2 translocates from the nucleus to the cytoplasm during cell cycle progression. We provide evidence of a novel role of *FANCD2* in ESCC tumor progression and its potential usefulness as a biomarker for ESCC disease management.

## 1. Introduction

Esophageal carcinoma (EC) ranks seventh in terms of incidence and was the sixth most lethal cancer worldwide in 2018 [1]. Esophageal squamous cell carcinoma (ESCC) arises from epithelial cells and is the major histologic form, comprising 60–70%, of all EC cases [2], while EC adenocarcinomas are the second major histological subtype. Despite the poor prognosis of ESCC, little is known about the drivers and targets for molecular therapy of this deadly cancer.

*Fanconi anemia complementation group D2* (*FANCD2*) encodes a protein required for the Fanconi anemia (FA) pathway, which is mono-ubiquitinated in response to DNA damage and essential for repairing DNA inter-strand crosslinks (ICLs). *FANCD2* deficiency in mice confers cancer susceptibility for acute myeloid leukemia and squamous cell carcinomas [3,4]. Published targeted next-generation sequencing (NGS) analyses show that germline *FANCD2* variants are associated with breast cancer [5,6] and head and neck squamous cell carcinoma (HNSCC) susceptibility [7]. These results suggest that germline *FANCD2* mutations increase cancer susceptibility. However, less is known about the wild-type (WT) *FANCD2* functional role in tumorigenesis. Overexpression of *FANCD2* is positively associated with tumor size and poor prognosis in breast cancer [8,9,10], ovarian cancer [11,12], nasopharyngeal carcinoma [13], glioblastoma [14], and endometrial carcinoma [15]. Little is known about its function in ESCC.

The aim of the current study is to evaluate the functional impact of FANCD2 protein expression in ESCC development using in vivo and in vitro functional assays, as well as to identify putative mechanisms. We examined the RNA expression of *FANCD2* in normal/ESCC paired tissue samples and found that *FANCD2* is significantly upregulated in tumors as compared to normal tissues. Consistently, FANCD2 protein is also overexpressed in ESCC cell lines. We demonstrated that *FANCD2* plays roles in ESCC development by regulating cell cycle progression. *FANCD2* promotes cell cycle progression by modulating cyclin proteins and checkpoint proteins, independent of its role in DNA damage repair. FANCD2 localizes to and is only mono-ubiquitinated in the nucleus. These results suggest that *FANCD2* confers a malignant phenotype in ESCC and may serve as a biomarker for ESCC therapeutics.

## 2. Materials and Methods

### 2.1. Clinical Specimens

Four pairs of ESCC patient tissues were collected from Queen Mary Hospital between 2001 and 2003, as previously reported [16]. Approval for this study was obtained from the Hospital Institutional Review Board at the University of Hong Kong (IRB UW-14-457).

### 2.2. RNA Sequence Analysis

We sequenced the RNA of four pairs of patient tissues using the Illumina HiSeq 2000 (2 × 100 bp paired reads). Three sets of public RNA sequencing (RNA-seq) data (SRP007169, SRP008496, SRP064894) were downloaded from the SRA database. All RNA-seq reads were aligned to reference genome hg19 using TopHat (version 2.0.14, bowtie version 2.2.4) [17]. The gene expression levels were calculated using Cufflinks (version 2.2.1) [18].

### 2.3. Cell Lines

An immortalized human esophageal epithelial cell line NE1 (Research resource identifier: CVCL_E306) and ESCC cell lines including KYSE30 (CVCL_1351), KYSE150 (CVCL_1348), and KYSE450 (CVCL_1353) were cultured as previously described [19]. KYSE30TSI was derived from a subcutaneous tumor established with KYSE30 [19]. KYSE150Luc is the KYSE150 labeled with firefly luciferase [20]. Cell line authentication by STR DNA profiling and mycoplasma test by PCR amplification of mycoplasma DNA were performed for all cell lines used.

### 2.4. Plasmids and Lentivirus Preparation and Infection

Clustered Regularly Interspaced Short Palindromic Repeat (CRISPR) systems were used with *FANCD2*-targeted sgRNA (sequence: AGAAGCTCTTTCAGACCCTG) to generate *FANCD2* knockout (KO) cell lines [11]. Non-targeting sgRNA (sequence: GTTCCGCGTTACATAACTTA) was used as a negative control [12]. The Renilla luciferase-POLIRES-Firefly luciferase cassette was cloned into pLVXEF1a [11]. Lentivirus preparation and infection were performed as described [19].

### 2.5. Western Blot Analysis

Cell protein lysates were electrophoresed on 4% SDS-PAGE gels for FANCD2 analysis and 12% SDS-PAGE gels for cell-cycle markers. Proteins were transferred to PVDF membrane, blocked, and incubated with primary antibodies as previously described [19]. Table 1 summarizes detailed information for the antibodies used against FANCD2, nuclear matrix protein p84, and all antibodies for cell cycle analysis from cyclin antibody sampler kit (#9869, Cell Signaling, MA, USA) and cell cycle regulation antibody sampler kit (#9932, Cell Signaling).

### 2.6. In Vivo Tumorigenicity Assay

Twelve female BALB/c athymic nude mice (6–8 weeks old) per group were used in subcutaneous injections and 16 mice per group were used in tail-vein injections. Subcutaneous injection was performed as previously described [19], with 1.25 × 10^6^ cells/site for KYSE30TSI, 2 × 10^6^ cells/site for KYSE150, and 5 × 10^6^ cells/site for KYSE450. Tail-vein injection was performed with 1 × 10^6^ of KYSE150Luc cells per mouse.

Mice were monitored for lung metastasis at 3, 4 and 5 weeks after tail-vein injection by bioluminescence live animal imaging using the PE IVIS Spectrum Image System (PerkinElmer, Waltham, MA, USA). Mice were anesthetized with fluanisone/fentanyl during imaging. Images were captured 15 min after mouse intraperitoneal injection of D-Luciferin potassium salt (Synchem, Felsberg-Altenburg, Germany) at 150 mg/kg.

For the survival study, mice were sacrificed when weights decreased to 16.0 g. All experimentation in the animals was performed in compliance with the protocols approved by the Committee on the Use of Live Animals in Teaching and Research of the University of Hong Kong (CULATR #3631-15).

### 2.7. MTT Assay

Cells were plated at a concentration of 2 × 10^3^ cells per well in 96-well plates for cell proliferation assays. The proliferation and viability of cells were determined by the 3-(4,5-dimethylthiazol-2-yl)-2,5-diphenyltetrazolium bromide (MTT) assay as previously described [9].

### 2.8. Colony Formation Assay

Cells were seeded in 6-well plates at a density of 2000 cells/well. After two-week culture, cells were fixed in 4% paraformaldehyde followed by 1 × Giemsa stain (Sigma Aldrich, St Louis, MO, USA). Excess Giemsa was removed by running water. Images were captured and cell colonies were counted using the Gel Doc XR system (Bio-Rad Laboratories, Hercules, CA, USA).

### 2.9. Synchronization and Flow Cytometry Analysis

For synchronization in G1/S phase, cells were seeded in 100 mm plates to reach ~10–20% confluence and treated with thymidine as previous published [13]. After release from blocks, cells were harvested by trypsinization and fixed in 70% (*v*/*v*) ethanol at 4 °C overnight. The fixed cells were rinsed with PBS and incubated in PBS containing 50 µg/mL propidium iodide (PI) and 0.1 mg/mL RNase A for 30 min at 37 °C in the dark. Cells were immediately analyzed on a BD FACSCantoII flow cytometer (Becton Dickinson, Franklin Lakes, NJ, USA). The cell cycle graph was analyzed using the Flowjo software (Becton Dickinson; version 10.6.2; https://www.flowjo.com/solutions/flowjo).

### 2.10. In Vitro Chemotherapy Treatment

Cells were seeded in 6-well plates at 2 × 10^5^ per well and treated with culture medium containing 2 µM cisplatin (Sigma Aldrich). Protein expression levels were assessed using Western blotting analysis after 24-h incubation.

Cells were seeded in 96-well plates at 6000 per well in culture medium containing cisplatin (0, 2.5, 5, 10, 20 µM) or mitomycin C (MMC; 0, 0.625, 1.25, 2.5, 5 µM; Sigma). Cisplatin/MMC sensitivity was assessed using the MTT assay after 72-h incubation.

### 2.11. Subcellular Fractionation

Subcellular fractionation was performed using the Subcellular Protein Fractionation Kit for Cultured Cells (Thermo Fisher Scientific, Buffalo, NY, USA) according to manufacturer’s protocol.

### 2.12. Statistical Analysis

Data are presented as the mean ± SD. Two-sided Student t test was applied unless indicated otherwise. The results were considered as statistically significant when the *p* value was less than 0.05.

## 3. Results

### 3.1. Upregulation of FANCD2 Gene Expression in ESCC Tissues and Cell Lines

**Figure 1:**
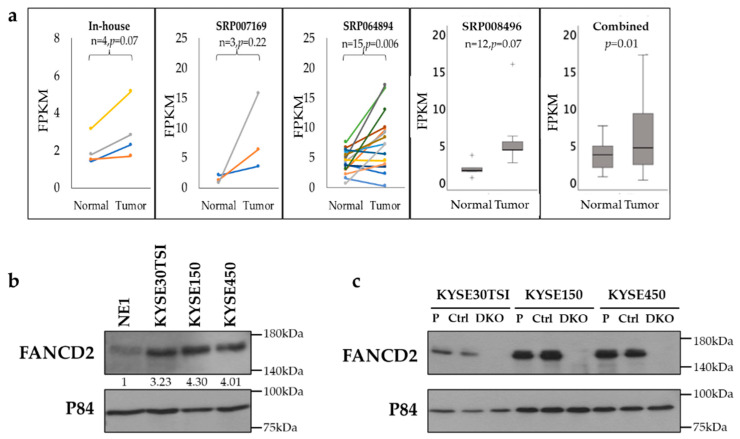
FANCD2 expression in ESCC tissues and cell lines. (**a**) *FANCD2* RNA expression was significantly upregulated in ESCC compared to the normal tissues using RNA sequencing and is presented as Fragments Per Kilobase Million (FPKM). +, outlier data. (**b**) FANCD2 protein expression was upregulated in three ESCC cell lines compared with the NE1 cell line. The numbers show the ratios of FANCD2 band intensity in ESCC cell lines relative to that in the NE1 cell line, normalized to the p84 loading. (**c**) Confirmation of endogenous FANCD2-KO in ESCC cell lines by Western blotting. P, parental; Ctrl, control; DKO, FANCD2 knockout; p84, loading control.

To determine the clinical significance of *FANCD2* in ESCC, *FANCD2* expression was examined in paired ESCC tumor and adjacent normal tissues by RNA-seq. *FANCD2* expression was upregulated in all four pairs of ESCC tumors as compared to adjacent normal esophageal epithelial tissues from our transcriptomic profiling analysis. In addition, *FANCD2* upregulation was also observed in three public datasets. The combination of all data together showed a significant upregulation of *FANCD2* RNA expression in ESCC tumor tissues (*p* = 0.01) (Figure 1a).

Consistently, FANCD2 protein expression was upregulated (>3-fold) in three ESCC cell lines (KYSE30TSI, KYSE150 and KYSE450) compared with the non-tumorigenic immortalized esophageal epithelial cell line NE1, as shown by Western blotting (Figure 1b and Figure S1). These data collectively are consistent with a role of FANCD2 conferring a malignant phenotype in ESCC development.

### 3.2. Depletion of FANCD2 Protein Expression Inhibits In Vivo Tumor Growth and Metastasis

**Figure 2:**
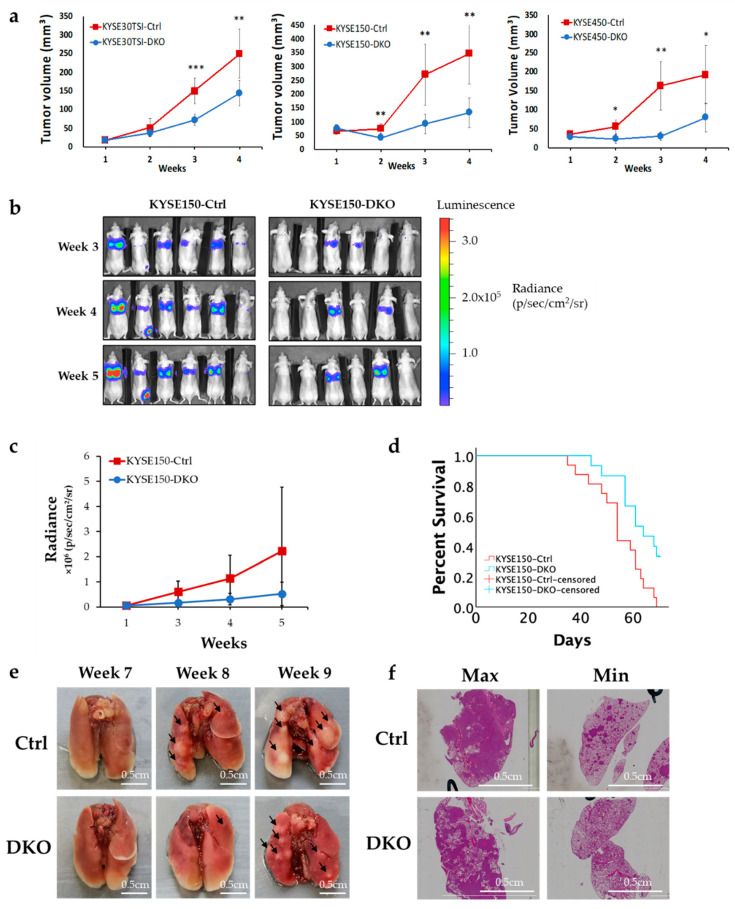
FANCD2 expression regulates in vivo tumor growth and metastasis. (**a**) Subcutaneous tumor growth is suppressed in FANCD2-KO ESCC cells. The data are shown as the mean ± 95% confidence interval (*n* = 12). (**b**) Representative images showing weaker and fewer bioluminescence signals in FANCD2-KO mice by live animal imaging. (**c**) Metastasis tumor volume was assessed at 3, 4 and 5 weeks after tail-vein injection. Using ROI analysis, tumor light intensity was calculated, which corresponds with the number of live cells in vivo. Lower bioluminescence signal was measured in FANCD2-KO mice compared with control mice. The data are shown as the mean ± 95% confidence interval (*n* = 16). (**d**) FANCD2-KO significantly increases the overall survival rate (Log Rank *p* = 0.007). (**e**) Representative lung images showing less and smaller tumor masses (arrow) in FANCD2-KO mice compared to the controls. (**f**) Representative lung sections showing fewer and smaller metastatic foci in FANCD2-KO mice under microscopy. Ctrl, control; DKO, FANCD2 knockout; * *p* < 0.05; ** *p* < 0.01; *** *p* < 0.005.

CRISPR-mediated FANCD2 protein expression depletion was performed in three ESCC cell lines resulting in almost complete knockout of FANCD2 (FANCD2-KO) protein expression (Figure 1c and Figure S2). To determine the role of *FANCD2* on ESCC primary tumor growth, the subcutaneous tumorigenicity nude mouse model was utilized. Significant suppression of tumor growth was observed in FANCD2-KO cells, as compared to control cells in all three cell lines tested (Figure 2a).

To determine the role of *FANCD2* on ESCC tumor metastasis, the tail-vein experimental metastasis model in nude mice was utilized using KYSE150Luc cells. An obvious reduction in lung metastasis formation was observed after three weeks in the FANCD2-KO group in contrast to the control group (Figure 2b,c). Mice bearing FANCD2-KO cells showed significantly better survival than the mice bearing control cells (Figure 2d; Log Rank *p* = 0.007). In addition, fewer and smaller metastases were observed in the lung sections of mice bearing FANCD2-KO cells (Figure 2e,f). These findings strongly indicate a role of *FANCD2* in supporting ESCC tumor development.

### 3.3. FANCD2-KO Suppresses In Vitro Cell Proliferation and Colony Formation in ESCC Cells

**Figure 3:**
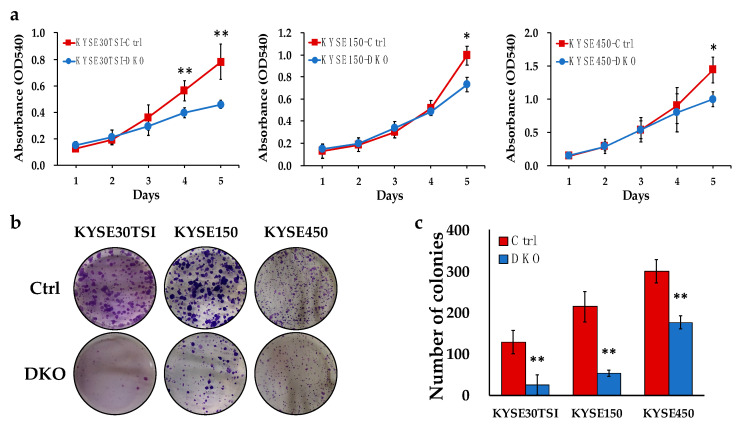
FANCD2 expression regulates in vitro cell proliferation and colony formation. (**a**) Cell proliferation was suppressed as assessed by MTT assay in FANCD2-KO ESCC cell lines. The data are shown as the mean ± 95% confidence interval (*n* = 3). (**b**) Representative images of colony formation assays are shown. (**c**) Colony formation ability was suppressed in FANCD2-KO ESCC cell lines. Data are representative of three independent experiments done in triplicates and expressed as the mean ± 95% confidence interval (*n* = 3). Ctrl, control; DKO, FANCD2 knockout; * *p* < 0.05; ** *p* < 0.01.

To dissect the functional impact of FANCD2-KO, in vitro cell proliferation assays were performed. Consistent with the in vivo data, FANCD2-KO cells showed suppressed cell proliferation in all three ESCC cell lines (Figure 3a). To investigate the role of FANCD2 on single cell-derived colony formation potential, an important factor for tumor metastasis, the clonogenic assay was performed. Consistently with the experimental metastasis data, FANCD2-KO cells formed significantly fewer and smaller colonies as compared to control cells (Figure 3b,c). These data verified the malignant phenotype of *FANCD2* on cell proliferation and colony formation in ESCC cell lines.

### 3.4. FANCD2-KO Suppresses Cell Cycle Progression in ESCC Cells

**Figure 4:**
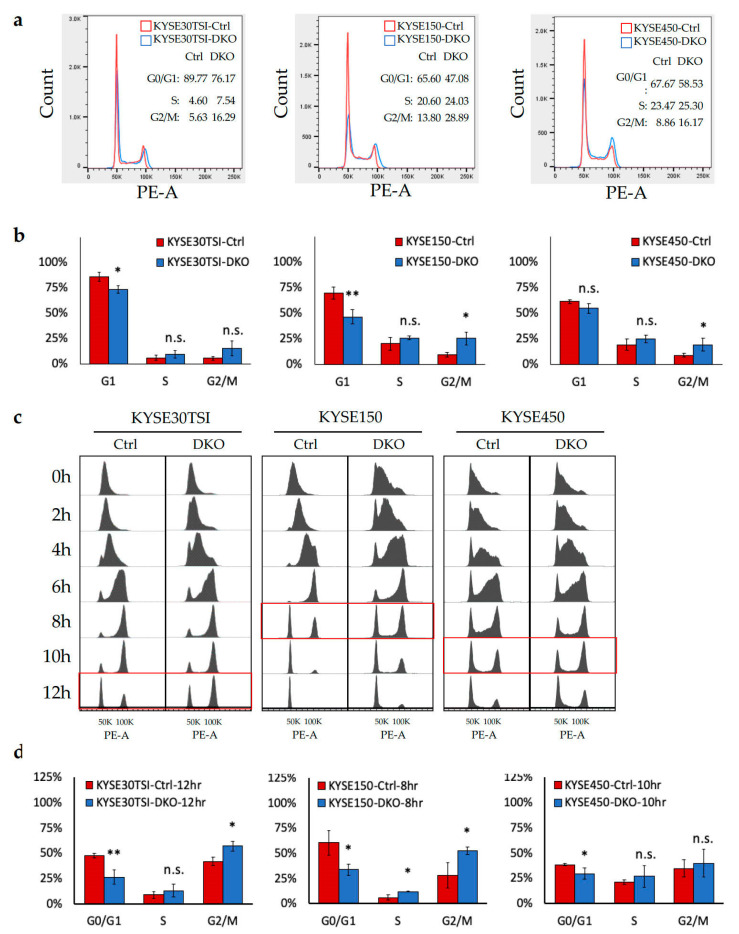
Analysis of cell cycle distribution and cell cycle progression in FANCD2-KO cells. (**a**) The proportion of cells in G1/S and G2/M phases changed in unsynchronized FANCD2-KO ESCC cell lines. Representative results are shown here. (**b**) Cell cycle profile analysis of three independent triplicate experiments expressed as the mean ± 95% confidence interval (*n* = 3). (**c**) FANCD2-KO postponed cell cycle progression after the release from the G1/S double-thymidine block. Cell cycle progression was determined by flow cytometric analysis of propidium iodide stained cells collected at the indicated time points (left). (**d**) Cell cycle profile analysis of the most significant different timepoints (highlighted with a red circle in Figure 4c) for three ESCC cell lines separately. Experiments were done in triplicates and expressed as the mean ± 95% confidence interval (*n* = 3). Ctrl, control; DKO, FANCD2 knockout; * *p* < 0.05; ** *p* < 0.01; n.s. *p* < 0.05.

Cell cycle profiles of FANCD2-KO cells were used to measure cellular DNA content. The percentage of cells in the G1 phase decreased and cells in G2/M phases slightly but constantly increased in all three unsynchronized ESCC cell lines (Figure 4a,b). After synchronization of the cells at early S phase before cell cycle profiling, FANCD2-KO cells progressed through the cell cycle more slowly than controls in three cell lines tested (Figure 4c,d). The disruption and delay of cell cycle progression suggest that FANCD2-KO suppresses ESCC cell proliferation by cell cycle regulation.

### 3.5. FANCD2-KO Affects G1/S Transition and Delays Entry to Mitosis in ESCC Cells

**Figure 5:**
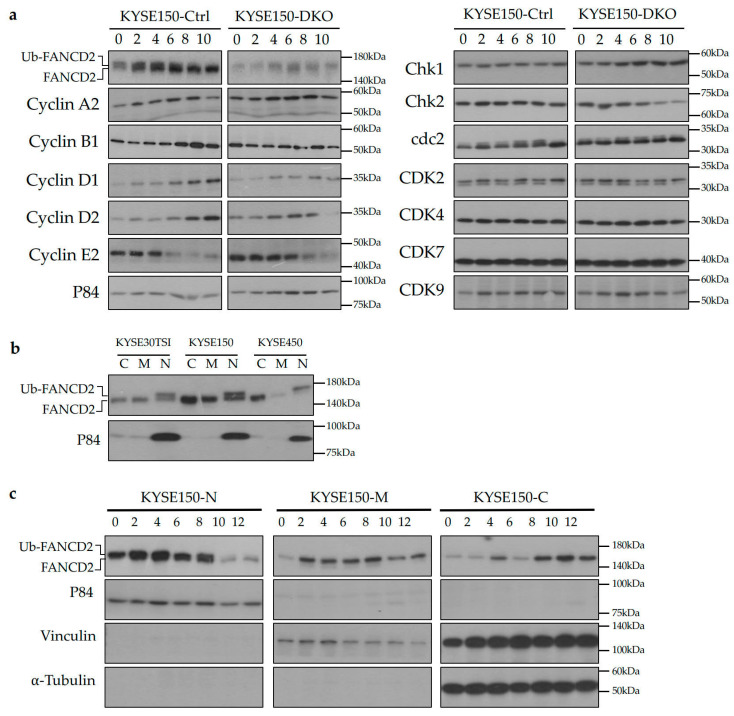
Analysis of cell cycle markers and *FANCD2* subcellular localization. (**a**) FANCD2-KO induced alterations in the expression of cyclin proteins that are essential for cell cycle progression. The protein lysates were collected at the indicated time points after double-thymidine block and analyzed by Western blotting. (**b**) Western blotting analysis showed mono-ubiquitination of FANCD2 only in the nucleus. (**c**) Western blotting analysis of FANCD2 protein in various cellular fractions at the indicated time points after double-thymidine block. N, nuclear fractions; M, membrane fractions; C, cytoplasmic fractions; Ctrl, control; DKO, FANCD2 knockout; p84, loading control; Vinculin, loading control; ⍺-Tubulin, loading control.

To investigate the molecular regulation of cell cycle progression, cells were synchronized at early S phase followed by examining expression of cell cycle regulators by Western blotting. The delayed degradation of cyclin E2 in FANCD2-KO cells suggests partial G1/S arrest (Figure 5a, Figures S3–S6). Subsequently, the delayed degradation of cyclin A2 and the lack of upregulation of cyclin B1 indicate delayed entry and exit of mitosis for FANCD2-KO cells (Figure 5a, Figures S3–S6). As cells further progressed through the cell cycle, FANCD2-KO cells showed inactivation of cyclin D1 and cyclin D2, which indicates delayed initiation of a new cell cycle round. Furthermore, Chk1 was upregulated and Chk2 was downregulated in FANCD2-KO cells, indicating that ATR and ATM pathways are involved in FANCD2-KO-induced suppression of cell cycle progression. These results are concordant with the flow cytometry analysis and suggests that *FANCD2* is necessary for accurate cell cycle progression in ESCC cells.

### 3.6. Ubiquitinated FANCD2 Localizes to the Nucleus to Regulate Cell Cycle Progression in ESCC Cells

The Western blotting analysis of synchronized cells shows that FANCD2 ubiquitination in the S phase and de-ubiquitination during G2/M phases (Figure 5a). Subcellular localization of FANCD2 was examined by cellular fractionation. The mono-ubiquitinated FANCD2 solely localized to the nucleus in an unsynchronized population of ESCC cells (Figure 5b and Figure S7). The upregulation of FANCD2 protein was shown in the nuclear fraction at first, and then in the membrane fraction, and finally in the cytoplasmic fraction (Figure 5c and Figure S8). The sole nuclear localization was also confirmed during the S phase in the synchronized ESCC cells (Figure 5c and Figure S8). This translocation and ubiquitination of FANCD2 protein indicate that it plays important roles in regulation of the ESCC cell cycle.

### 3.7. FANCD2 Is Likely Dispensable for DNA Damage Repair in ESCC Cells

**Figure 6:**
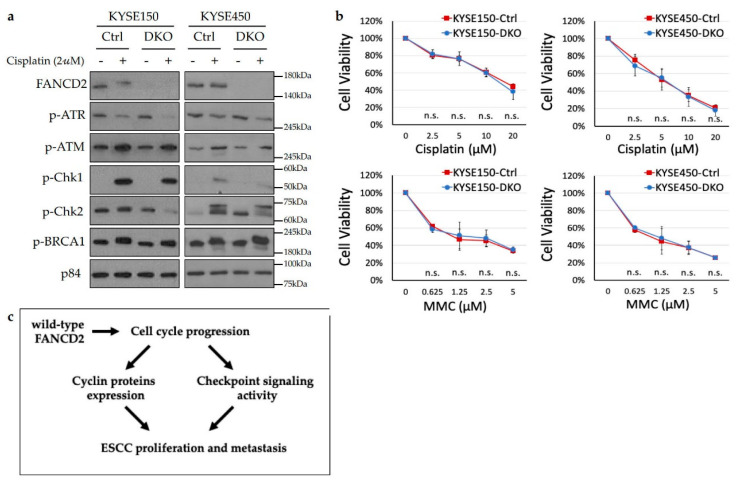
Analysis and schematic diagram of *FANCD2* functions. (**a**) Western blotting analysis of checkpoint proteins in response to 2µM cisplatin treatment. (**b**) MTT assay on cells treated with cisplatin or MMC for 72 h. The data are shown as the mean ± 95% confidence interval (*n* = 3). (**c**) Schematic diagram of FANCD2 conferring a malignant phenotype in ESCC. Ctrl, control; DKO, FANCD2 knockout; n.s., *p* < 0.05.

Cells were treated with cisplatin or MMC to further investigate how *FANCD2* functions in DNA damage repair in ESCC. Cisplatin treatment increased FANCD2 mono-ubiquitination and phosphorylation of ATR (p-ATR), ATM (p-ATM), Chk1 (p-Chk1), Chk2 (p-Chk2) and BRCA1 (p-BRCA1) (Figure 6a, Figures S9 and S10), while FANCD2-KO suppressed p-ATR and p-Chk2 in response to DNA damage (Figure 6a, Figures S9 and S10). The FANCD2 expression level does not affect chemosensitivity to cisplatin and MMC in ESCC cells (Figure 6b). These results indicate that *FANCD2* is likely dispensable in the DNA damage repair response to cisplatin or MMC in ESCC cells.

## 4. Discussion

*FANCD2* has been well-studied in regards to cancer susceptibility and initiation. *FANCD2*, the pivotal player in the FA/BRCA repair pathway, is important for maintaining genome stability in response to a variety of genotoxic stresses [14,15]. Mono-ubiquitinated FANCD2 is often portrayed as the functional representative of activated FA signaling [16,17]. Biallelic germline mutations in *FANCD2* increase cancer susceptibility [18]. However, less is known about its functional influence in tumorigenesis. Tissue microarray analysis showed that upregulation of *FANCD2* is positively associated with tumor size and adverse prognosis in breast cancer [8,9,10], ovarian cancer [11,12], nasopharyngeal carcinoma [13], glioblastoma [14], and endometrial carcinoma [15]. Our analysis of in-house and publicly available ESCC transcriptome datasets showed that *FANCD2* is significantly upregulated in ESCC tumors. We also showed that FANCD2 protein expression is consistently upregulated in ESCC cells. Given the low frequency of somatic *FANCD2* mutations in ESCC, the majority of upregulated *FANCD2* possesses WT sequences and are expected to exert WT functions [19,20]. The current study suggests that function of WT *FANCD2* is favored in ESCC; we further provide functional evidence for this using various in vivo and in vitro assays, which are consistent in showing that high-*FANCD2* expression is associated with increased risk of metastasis and poor prognosis in ESCC. Our study is the first functional analysis suggesting that *FANCD2* confers a malignant phenotype and may be used as a novel prognostic marker for ESCC. However, future overexpression experiments in normal esophageal epithelial cell lines are needed to clarify whether the malignant phenotype of FANCD2 overexpression is only present in transformed cells and, therefore, reflects a late step in cancer evolution of ESCC.

We showed by cell cycle analysis that the tumor-suppressive effect of FANCD2-KO in ESCC was mediated by suppression of cell proliferation through suppression of cell cycle progression. *FANCD2* plays roles in nearly all phases of cell cycle regulation. In the G1/S phases, *FANCD2* is required for the full activation of DNA replication by participating in initiation of DNA replication [21] and prevention of replication forks together with *RAD51-BRCA1/2* [22]. The intra-S phase *ATR-Chk1* checkpoint promotes FANCD2 mono-ubiquitination and subnuclear foci assemble in response to DNA damage [23], whereas *FANCD2* deficiency leads to a *Chk1*-dependent G2 accumulation [24,25]. Subsequently, *USP1* deubiquitinates FANCD2 to recycle FANCD2 and promote S phase exit [26]. In the G2/M phases, the FANCD2/FANCI protein dimers interlinking with sister chromatids through BLM-associated ultra-fine DNA bridges (UFBs) are responsible for proper chromosome segregation independent of the downstream FA pathway and *ATM/ATR* checkpoint [27,28]. *FANCD2* is also required for proper phosphorylation of H2AX and, hence, activation of the *ATM-Chk2* checkpoint [25,29]. Considering the abnormal expression of Chk1 and Chk2 in FANCD2-KO ESCC cells and their crucial roles in cell cycle regulation, *FANCD2* likely regulates cell cycle progression by modulating checkpoint signaling.

Despite our data suggesting that *FANCD2* plays roles in repair of cisplatin-induced DNA damage, FANCD2-KO cells did not show enhanced chemosensitivity to cisplatin and MMC treatments. The FA/BRCA2 pathway is critical for the orchestration of the cellular response to cisplatin and MMC, two DNA cross-linking agents, while *FANCD2* deficiency increased chemosensitivity to cisplatin and MMC treatments in HeLa cells [23,30]. However, this was not observed in our study, implying that *FANCD2* is dispensable for repairing cisplatin/MMC-induced DNA damage. It is likely that DNA repair proceeds through alternate pathways in ESCC. Further in-depth study is needed to elucidate the mechanism behind this.

Our studies identified *FANCD2* subcellular localization in synchronized ESCC cells to better understand its function in cell cycle regulation. Reports indicate that nuclear concentration and mono-ubiquitination of *FANCD2* to form *FANCD2/FANCI* nuclear foci and co-localization with *BRCA1* are required for the activation of the FA pathway in cellular response to both intra- and extra-cellular DNA damage in the S phase [31,32]. After DNA repair is completed, a subset of FANCD2 proteins is excluded from the chromosomes and relocates to the cytoplasm at the onset of mitosis [33]. The remaining nuclear FANCD2 on the sister chromatids in G2/early mitosis (prophase and metaphase) and subsequent resolution at anaphase are critical for proper chromosome segregation and prevention of micro-nucleation [27,33]. Our subcellular location study clearly shows the nuclear localization of mono-ubiquitinated FANCD2 in S/G2/early M phases and cytoplasm translocation during mitosis, supporting the multiple roles *FANCD2* plays in cell cycle regulation in ESCC cells. The membrane localization of FANCD2 is possibly associated with its functions in mitochondria and regulation of ATP metabolism [34,35]. The cellular ATP concentration varies through the cell cycle, reaching a peak at G2/M- and a minimum at late G1/early S phase [36], which is consistent with our observed *FANCD2* expression levels in membrane fractions.

## 5. Conclusions

In summary, the current study is the first functional study exploring a *FANCD2* malignant phenotype in ESCC development, through regulating cell cycle progression (Figure 6c), suggesting that *FANCD2* may serve as a prognostic biomarker and potential drug target in ESCC management. These observations also provide new evidence for an interplay between cancer-associated signaling pathways and cell cycle regulation.

## Figures and Tables

**Table 1:** Antibody information Used for Western Blots.

Antibody	Company	Catalog Number	Dilution	Antibody	Company	Catalog Number	Dilution
FANCD2	Santa Cruz	sc20022	1:1000	CDK4	Cell Signaling	12,790	1:1000
p84	GeneTex	GTX70220	1:2000	CDK7	Cell Signaling	2090	1:1000
Cyclin A2	Cell Signaling	4656	1:1000	CDK9	Cell Signaling	2316	1:1000
Cyclin B1	Cell Signaling	4138	1:1000	p-ATR	Cell Signaling	2853	1:1000
Cyclin D1	Cell Signaling	2978	1:1000	p-ATM	Cell Signaling	13,050	1:1000
Cyclin D2	Cell Signaling	3741	1:1000	p-Chk1	Cell Signaling	12,302	1:1000
Cyclin E2	Cell Signaling	4132	1:1000	p-Chk2	Cell Signaling	2661	1:1000
Chk1	Cell Signaling	2345	1:500	p-BRCA1	Cell Signaling	9009	1:1000
Chk2	Cell Signaling	3440	1:1000	Vinculin	Cell Signaling	13,901	1:2000
cdc2	Cell Signaling	28,439	1:1000	⍺-Tubulin	Cell Signaling	2125	1:2000
CDK2	Cell Signaling	2546	1:1000				

## Supplementary Materials

The following are available online at https://www.mdpi.com/2072-6694/12/9/2545/s1, Figure S1: Supplements for Figure 1b, Figure S2: Supplements for Figure 1c, Figure S3: Whole length western blots of western blots shown in Figure 5a, Figure S4: Fold change of densitometry readings/intensity ratios of western blots shown in Figure 5a, Figure S5: Whole length western blots of western blots shown in Figure 5a (continued), Figure S6: Fold change of densitometry readings/intensity ratios western blots shown in Figure 5a (continued), Figure S7: Supplements for Figure 5b, Figure S8: Supplement for Figure 5c, Figure S9: Supplements for Figure 6a, Figure S10: Supplements for Figure 6a (continued).
