# Supplementary material for: FANCD2 Confers a Malignant Phenotype in Esophageal Squamous Cell Carcinoma by Regulating Cell Cycle Progression
Source: Cancers (Basel). 2020 Sep 7;12(9):2545. doi: 10.3390/cancers12092545 (PMC7565464; doi:10.3390/cancers12092545)
Supplement: Supplementary file 1 [file cancers-12-02545-s001.pdf]

Supplementary Materials:

FANCD2 confers a malignant phenotype in esophageal squamous cell carcinoma by regulating cell cycle progression

Lisa Chan LEI, Valen Zhuoyou YU, Josephine Mun Yee KO, Lvwen NING, Maria Li LUNG

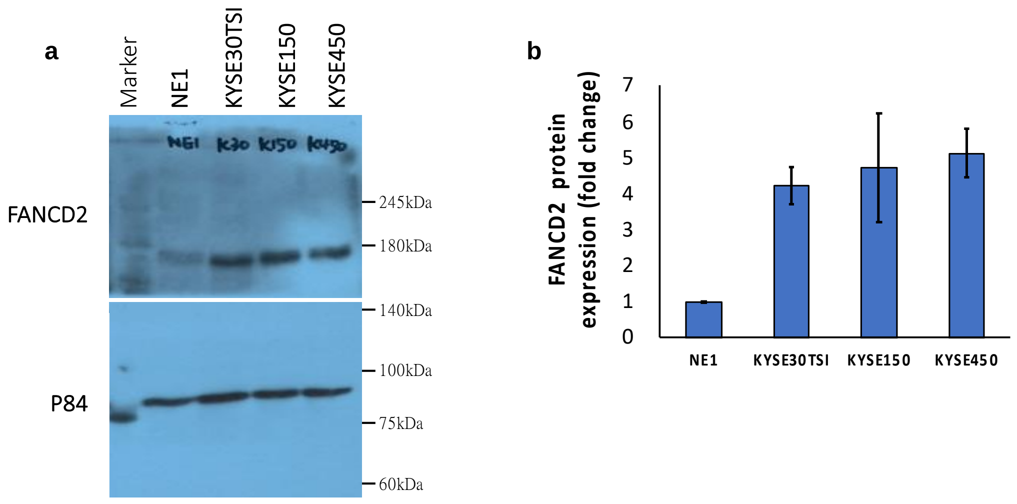

Figure S1. Supplements for Figure 1b. (a) Whole length western blots (of western blots shown in Figure 1b). (b) Fold change of densitometry readings/intensity ratios (normalized with background and p84, n=3). Error bar represents 95% confidence interval.

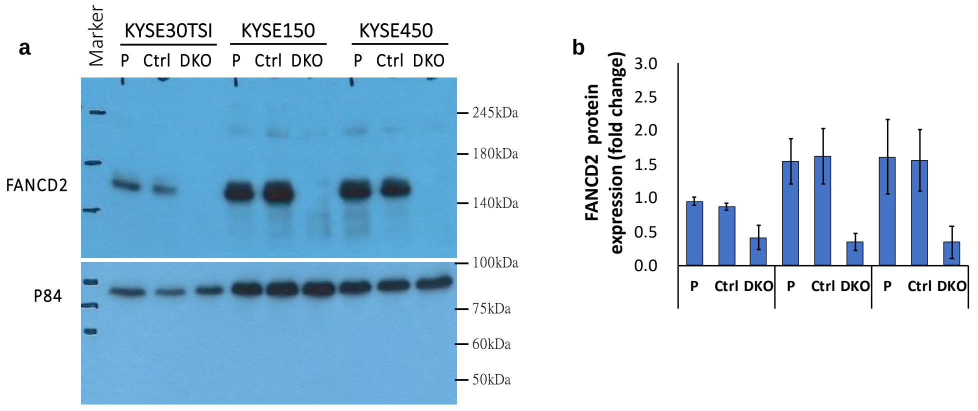

Figure S2. Supplements for Figure 1c. (a) Whole length western blots (of western blots shown in Figure 1c). (b) Fold change of densitometry readings/intensity ratios (normalized with background and p84, n=3). P, parental; Ctrl, control; DKO, FANCD2 knockout. Error bar represents 95% confidence interval.

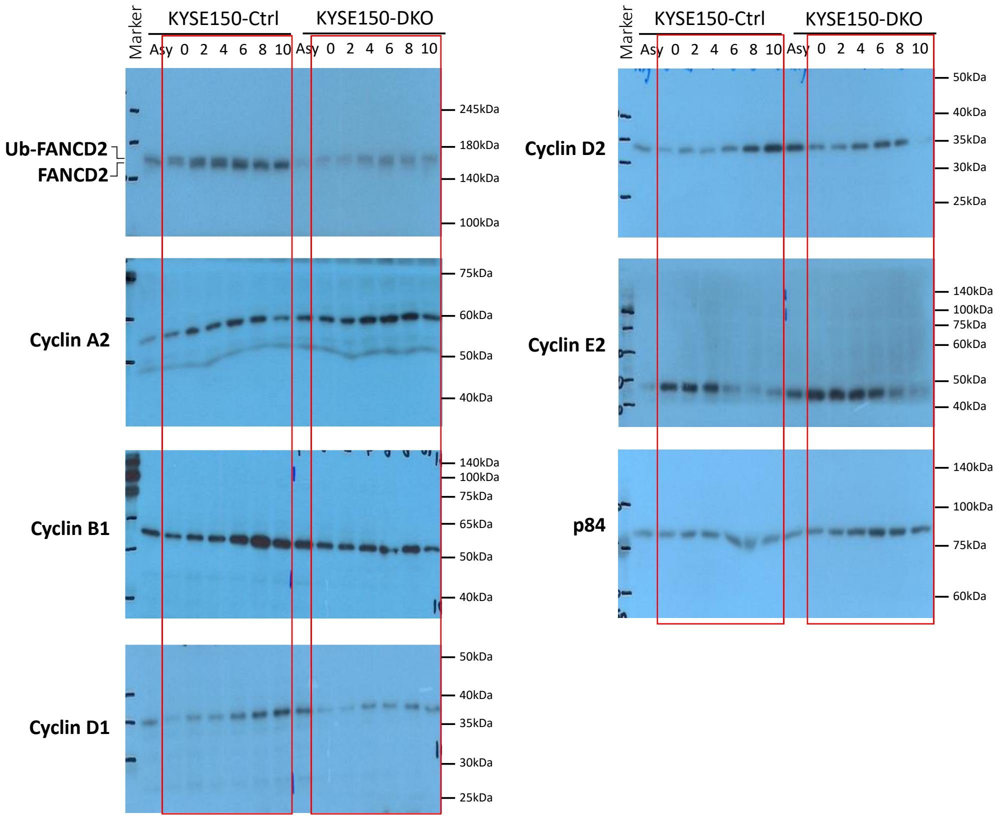

Figure S3. Whole length western blots (of western blots shown in Figure 5a). Asy, Asynchronized; Ctrl, control; DKO, FANCD2 knockout.

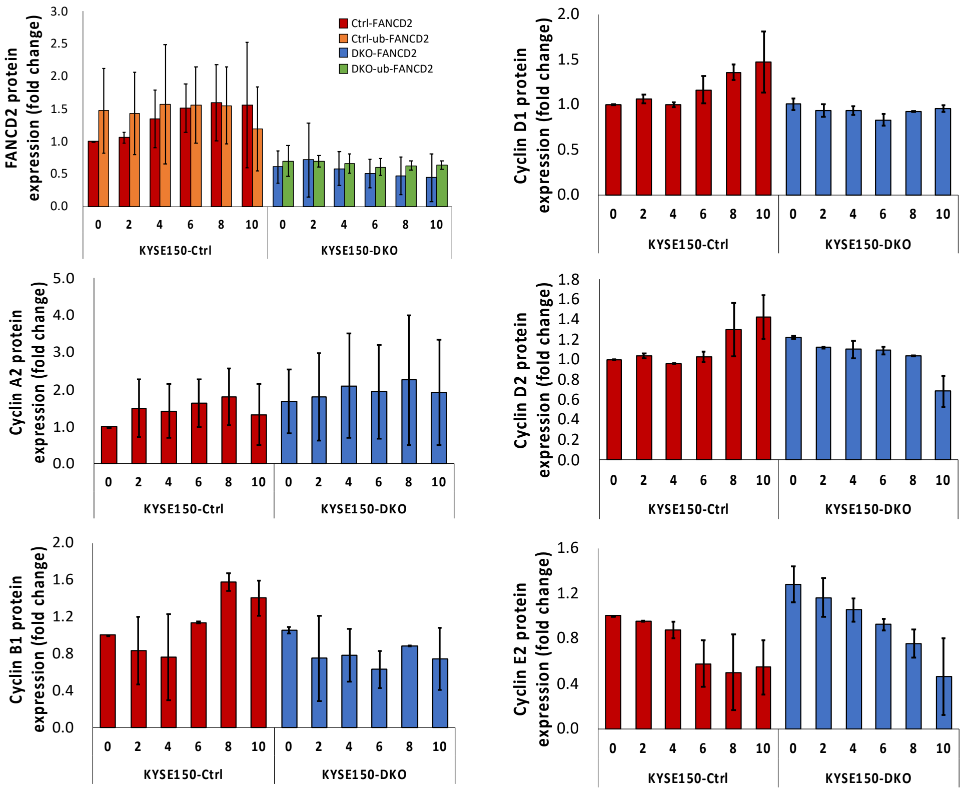

Figure S4. Fold change of densitometry readings/intensity ratios of western blots shown in Figure 5a (normalized with background and p84, n=2). Asy, Asynchronized; Ctrl, control; DKO, FANCD2 knockout. Error bar represents 95% confidence interval.

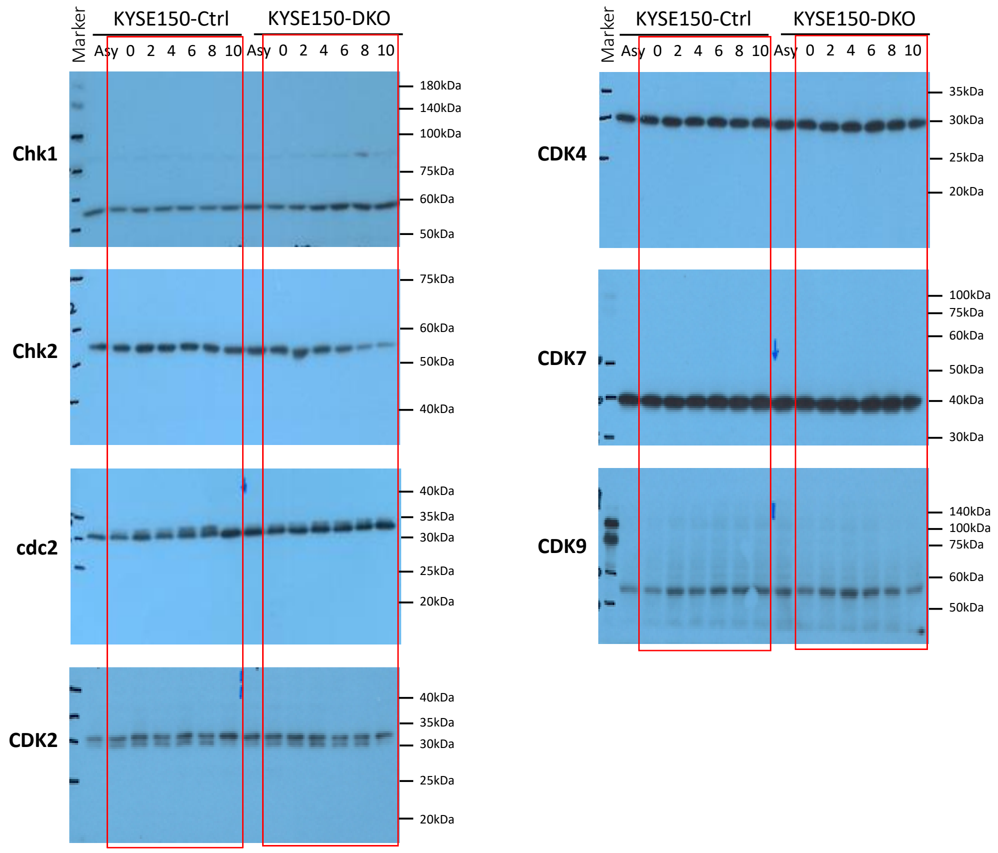

Figure S5. Whole length western blots (of western blots shown in Figure 5a, continued). Asy, Asynchronized; Ctrl, control; DKO, FANCD2 knockout.

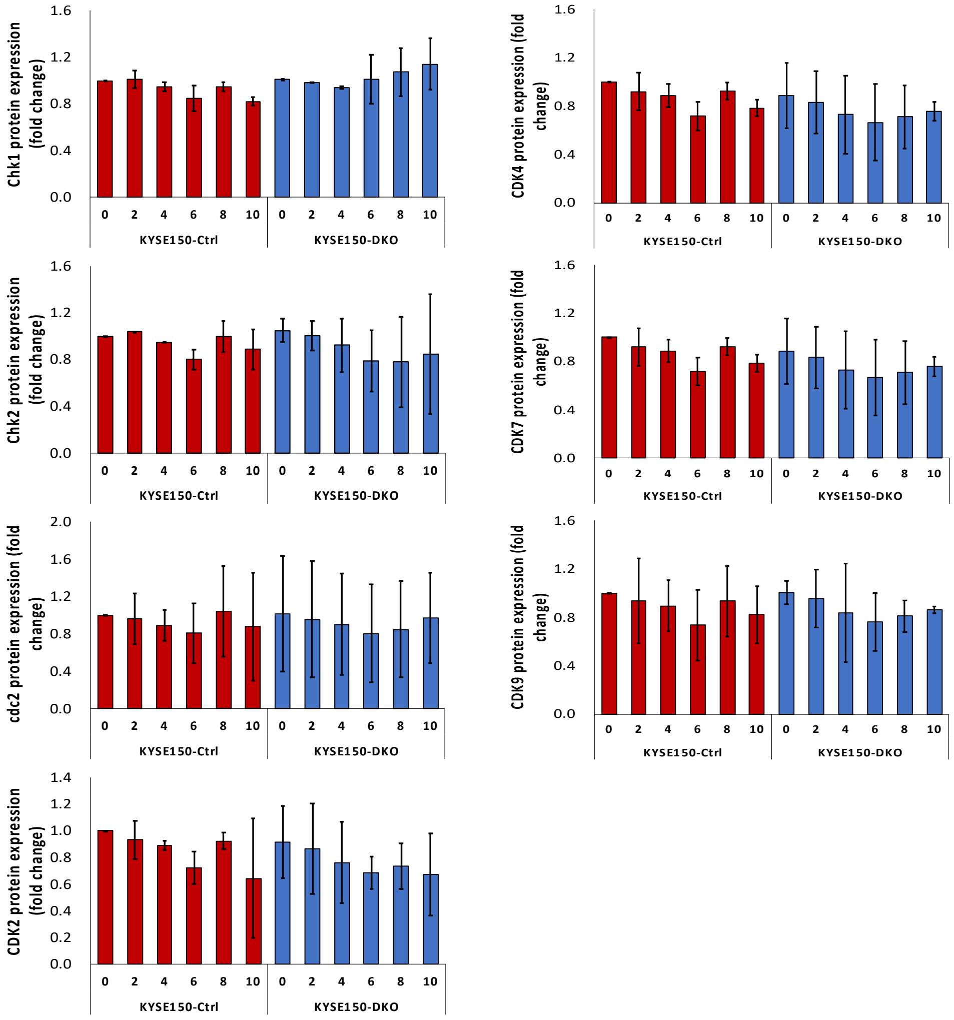

Figure S6. Fold change of densitometry readings/intensity ratios western blots shown in Figure 5a (continued, normalized with background and p84, n=2). Asy, Asynchronized; Ctrl, control; DKO, FANCD2 knockout. Error bar represents 95% confidence interval.

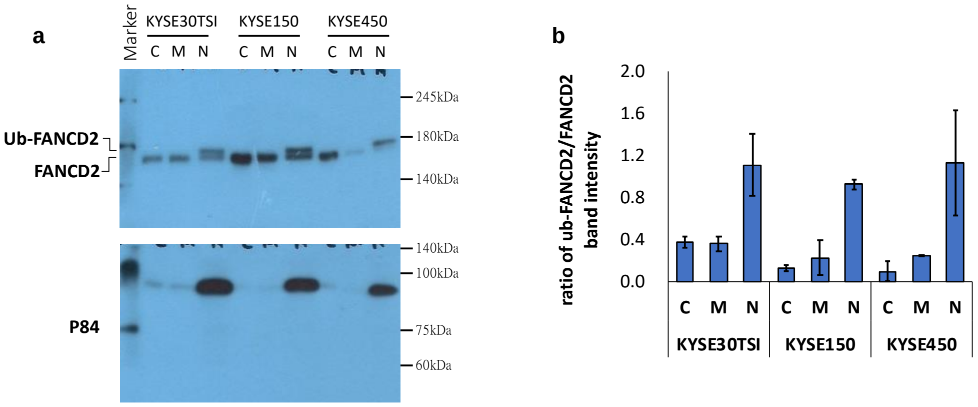

Figure S7. Supplements of Figure 5b. (a) Whole length western blots (of western blots shown in Figure 5b). (b) Ratio of ub-FANCD2/FANCD2 band intensity (normalized with background, n=2). C, cytoplasmic fractions; M, membrane fractions; N, nuclear fractions. Error bar represents 95% confidence interval.

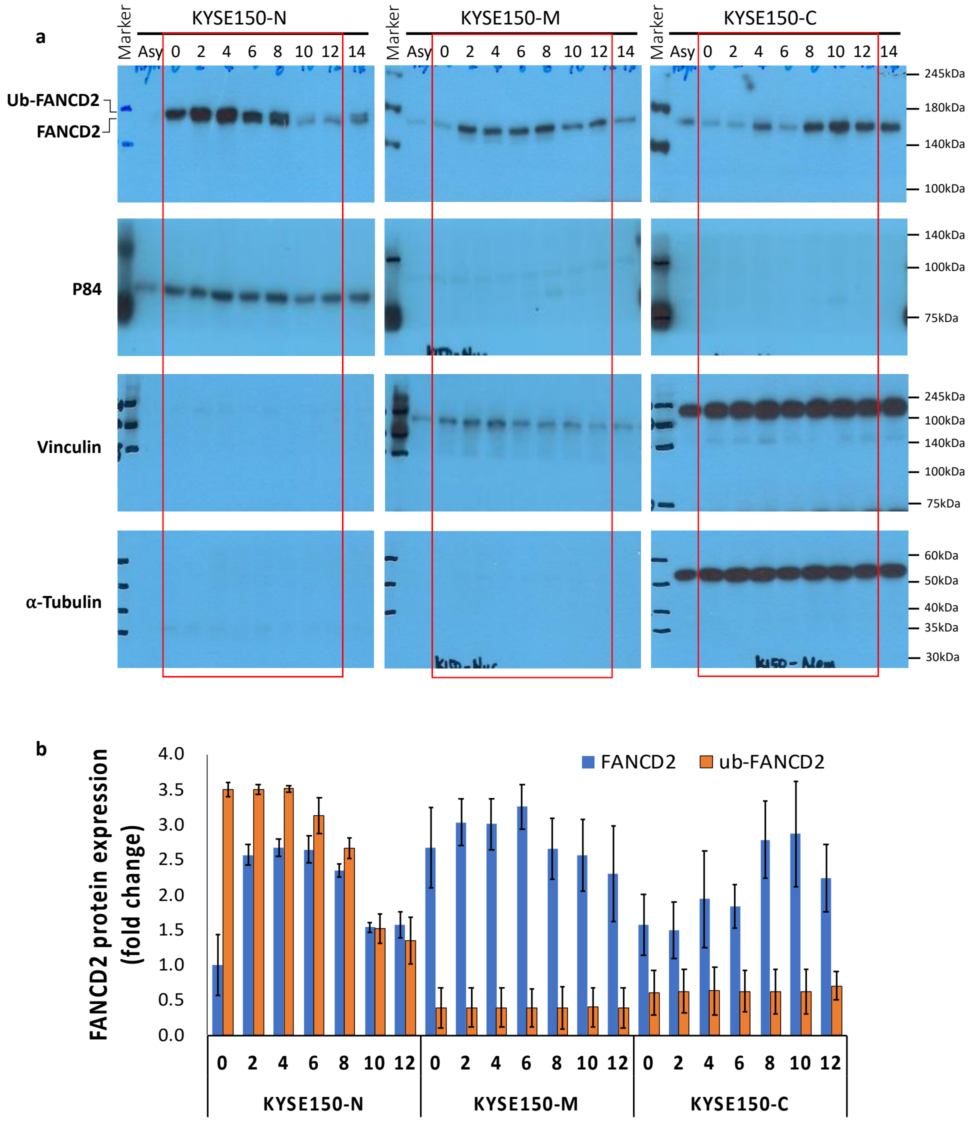

Figure S8. Supplement of Figure 5c. (a) Whole length western blots (of western blots shown in Figure 5c). (b) Fold change of FANCD2 protein expression (normalized with background and control, n=2). Asy, Asynchronized; N, nuclear fractions; M, membrane fractions; C, cytoplasmic fractions. Error bar represents 95% confidence interval.

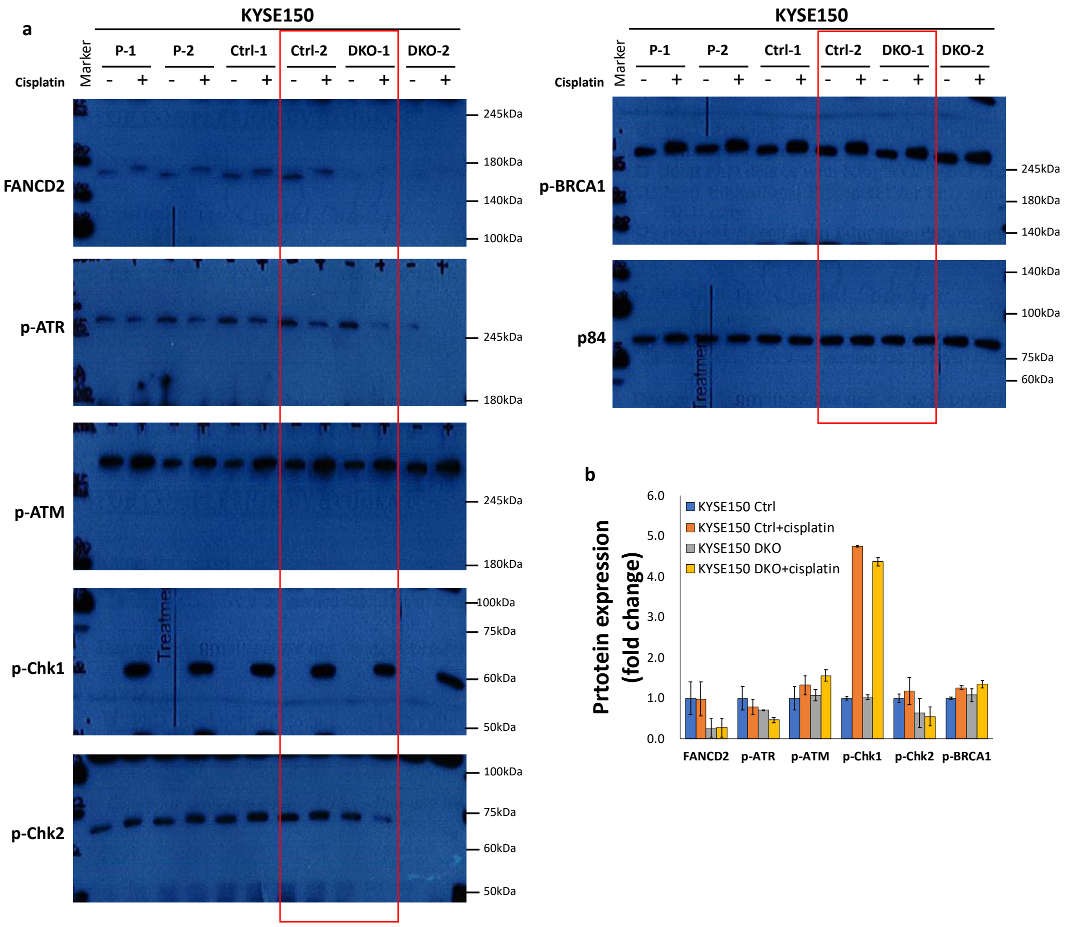

Figure S9. Supplements of Figure 6a. (a) Whole length western blots (of western blots shown in Figure 6a). (b) Fold change of densitometry readings/intensity ratios (normalized with background and control, n=2). P, parental; Ctrl, control; DKO, FANCD2 knockout. Error bar represents 95% confidence interval.

Note: To achieve comparable results, these Western blots were cut prior to probing for multiple antibodies to detect the distinct MW proteins. The antibodies used are all well-known to achieve clean bands. Blots in the same experiment were detected from the same batch of samples.

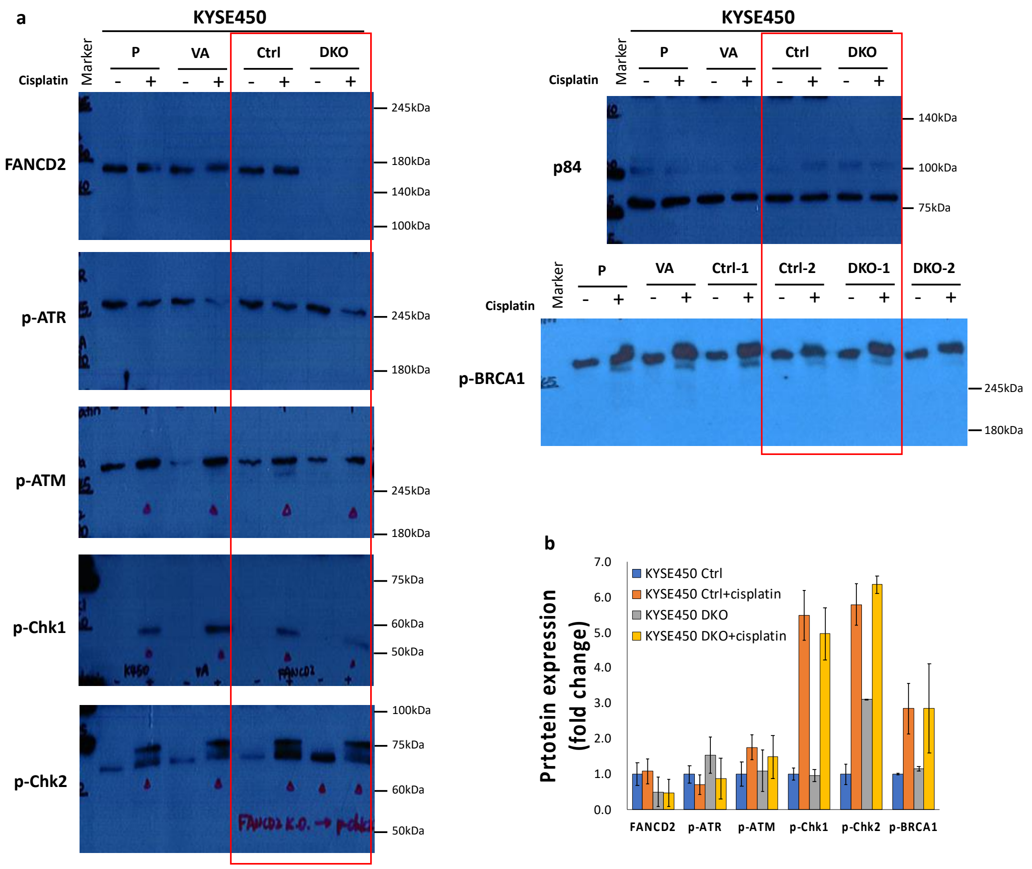

Figure S10. Supplements of Figure 6a, continued. (a) Whole length western blots (of western blots shown in Figure 6a, continued). (b) Fold change of densitometry readings/intensity ratios (normalized with background and control, n=2). P, parental; VA, vector alone; Ctrl, control; DKO, FANCD2 knockout. Error bar represents 95% confidence interval.

Note: To achieve comparable results, these Western blots were cut prior to probing for multiple antibodies to detect the distinct MW proteins. The antibodies used are all well-known to achieve clean bands. Blots in the same experiment were detected from the same batch of samples.
